# A self-assembled plasmonic optical fiber nanoprobe for label-free biosensing

**DOI:** 10.1038/s41598-019-43781-8

**Published:** 2019-05-14

**Authors:** Yuzhang Liang, Zhiyong Yu, Lixia Li, Ting Xu

**Affiliations:** 1 National Laboratory of Solid State Microstructures, College of Engineering and Applied Sciences, Collaborative Innovation Center of Advanced Microstructures, Key Laboratory of Intelligent Optical Sensing and Manipulation, Nanjing University, Nanjing, 210093 China; 2 College of Physics and Material Science, Henan Normal University, Xinxiang, 453007 China

**Keywords:** Biosensors, Metamaterials

## Abstract

The plasmonic optical fiber sensors have attracted wide attention for label-free biosensing application because of their high integration, small footprint and point-of-care measurement. However, the integration of plasmonic nanostructures on optical fiber probes always relies on the top-down nanofabrication approaches, which have several inherent shortcomings, including high cost, time-consuming, and low yields. Here, we develop a plasmonic nanohole-patterned multimode optical fiber probe by self-assembly nanosphere lithography technique with low fabrication cost and high yields. The multimode optical fiber possesses large facet area and high numerical aperture, which not only simplifies fabrication process, but also increases coupling efficiency of incident light. Originating from the resonant coupling of plasmonic modes, the plasmonic fiber nanoprobe has a distinct reflection dip in the spectrum and exhibits strong near-field electromagnetic enhancement. We experimentally investigate the sensing performances of plasmonic fiber nanoprobe, and further demonstrate it in real-time monitoring specific binding of protein molecules. The experimental results imply that the nanohole-patterned multimode optical fiber probe is a good candidate for developing miniaturized and portable biosensing systems.

## Introduction

Plasmonic nanostructure sensors, as a powerful analytical tool, have great application potentials in the fields of clinical diagnosis, drug development, and healthcare monitoring^1–10^. However, most of current nanostructure sensors are performed on planar substrates and tests rely on bulky optical microscope systems, which restricts the utilization of these sensors for point-of-care (POC) diagnostics and *in-vivo* measurements at specific location. Therefore, a miniaturized and portable plasmonic nanostructure sensor is highly desirable for practical applications. Optical fiber has unique properties of light-weight, small-size, flexibility, and can guide light to a remote location, which makes it be an attractive integrated platform for plasmonic nanostructures. Many efforts have been focused on the integration of nanostructures on optical fiber^11,12^, leading to the development of a novel and intriguing technology known as “Lab-on-Fiber”^13–15^. To date, several nanofabrication technologies established for planar substrates have been employed for the integration of plasmonic nanostructures on optical fiber tip, including electron beam lithography (EBL)^16–18^, focused ion beam (FIB) milling^19–21^, nanoimprint lithography^22,23^, and template transfer^24–26^. However, these top-down technologies suffer from several inherent shortcomings, such as high equipment cost, time-consuming and low yields. To overcome these drawbacks, nanosphere lithography technique, with unique features of rapid and large-scale preparation of ordered nanostructure array, is recently used to fabricate the nano-patterned fiber probe^27,28^. This technology enables the effective integration of various nanostructures onto optical fiber with low-cost and facile fabrication. However, the sensing probe fabricated by this technology so far is only applied for standard single mode optical fiber and surface enhancement Raman scattering measurements.

In this paper, based on self-assembly nanosphere lithography approach combined with plasmon etching, we develop a miniaturized and portable biosensing platform. Au nanoholes array as plasmonic sensing element is integrated on the tip of multimode optical fiber. The fabricated fiber nanoprobe shows a distinct reflection dip in the spectrum originating from the resonant coupling of plasmonic modes and is sensitive to environment around the probe surface. We experimentally investigate the sensing performances of plasmonic fiber nanoprobe, including bulk refractive index sensitivity and surface sensitivity, and the results agree well with theoretical predictions. In addition, due to high surface sensitivity, specific binding of protein molecules is experimentally monitored in real-time by plasmonic fiber nanoprobe.

## Results and Discussion

**Figure 1:**
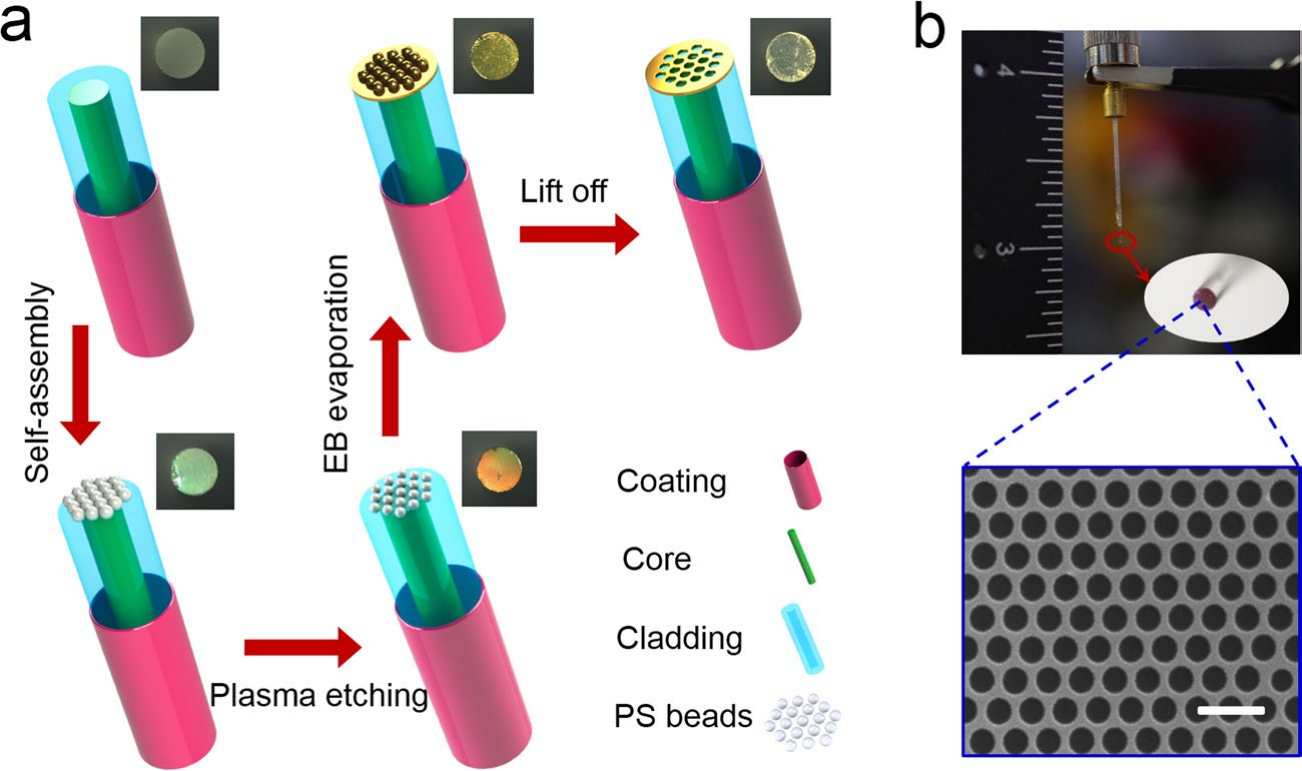
(**a**) Schematic of the bottom-up fabrication procedure for nanoholes array-based optical fiber probe. The entire procedure is divided into five sections: polishing optical fiber tip, PS nanosphere self-assembled on optical fiber tip, PS nanosphere etched by oxygen plasma, E-beam evaporating Au layer on optical fiber tip, PS nanosphere removed using organic solvent of tetrahydrofuran. Optical microscope images of fiber tip for each experiment section is shown in the top right corner. (**b**) A photograph of the optical fiber sensing probe. The enlarged inset is a SEM image of Au nanohole array. Scale bar, 1μm in the inset of (**b**).

Figure 1(a) summarizes fabrication procedure to prepare nano-patterned optical fiber nanoprobe using self-assembly nanosphere lithography. First, two end faces of a 45-mm-long multimode silica optical fiber is polished using a fiber polishing machine with 9μm, 3μm, 1μm, 0.03μm grit emery papers. The optical fiber has a core/cladding diameter of 400/425μm and numerical aperture (NA) of 0.22. Second, the self-assembly polystyrene (PS) nanosphere (500 nm diameter) monolayer at the air/water interface is transferred from water surface onto optical fiber tip. Here, the multimode optical fiber has a wider exposed surface than the standard single mode optical fiber (the core/cladding diameter: 9/125μm), which overcomes the problem of small facet area of single mode optical fiber which is difficult to transfer PS nanosphere monolayer without damaging it^27–29^. Third, PS nanosphere is etched by using common ION Wave 10 plasma system. The diameter of PS nanosphere can be controlled by adjusting etching time. When the etching time is set as 20 s, the diameter of PS sphere is reduced to about 300 nm. Next, a 5-nm-thick Ti adhesion film and 80-nm-thick Au film is deposited onto the nanosphere-patterned optical fiber tip by e-beam evaporation. Finally, the optical fiber probe is immersed into tetrahydrofuran solution and sonicated in an ultrasound bath for 8 minutes to selectively remove the nanosphere and leave Au nanohole array on the optical fiber tip. In above fabrication process, a large number of optical fiber nanoprobes could be fabricated simultaneously though plasma etching and vacuum deposition of metals, which greatly reduce fabrication time and cost. Optical microscope images of optical fiber tip for each step of fabrication process are also given in the Fig. 1(a). It can be clearly seen that optical fiber tip shows different colors after each experimental treatment. The photograph of fabricated Au nanohole array-based optical fiber sensing probe is shown in Fig. 1(b). The enlarged photograph of optical fiber tip confirms high quality of plasmonic structure covering entire optical fiber tip without large defects. The scanning electron microscope (SEM) image of Au nanohole arrays on optical fiber tip is shown in the inset of Fig. 1(b). The fabricated Au hexagonal nanohole array with high uniformity and fidelity has hole diameter of ~300 nm and periodicity of 500 nm.

**Figure 2:**
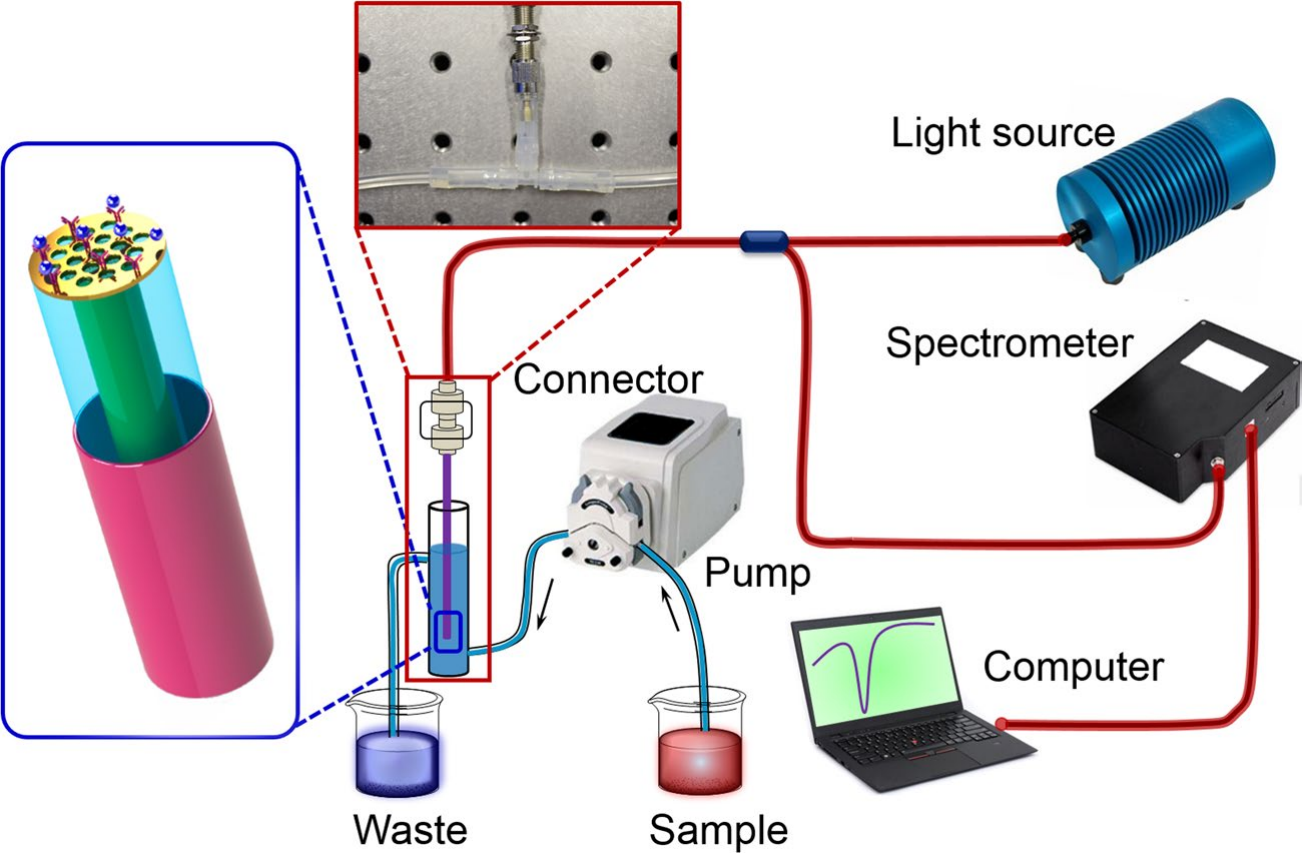
Schematic diagram of experiment setup for evaluating sensing performance of the plasmonic fiber nanoprobe.

The experimental configuration for sensing performance test of the nano-patterned optical fiber probe is shown in Fig. 2. Light source, spectrometer, and fiber probe are connected by a bifurcated optical fiber jumper made of multimode fiber (same as the probe), which is considered as the terminated reflection-type. The probe is mounted in the combined end of the fiber jumper thought the SMA905 connector and can be integrated with microfluidic flow-cell. A white light (360~2400 nm) from the halogen lamp (HL-2000-FHSA, Ocean Optics, Inc.) is coupled from one end of fiber jumper to the sensing probe. Compared to previous single mode optical fiber sensors^16,17,24,25,27,28^, here multimode optical fiber in this experiment have many advantages: First, the large facet area of multimode optical fiber can directly collect PS nanosphere without using the ceramic ferrule; Second, multimode optical fiber increases coupling efficiency of incident light. Without using high power laser, inexpensive light emitting diode and the halogen lamp is good enough to satisfy the testing requirements of multimode optical fiber, which can further reduce cost and facilitate system to integrate; Third, multimode optical fiber decreases the difficulty of handling in fabricated process. The reflection spectrum is collected at the other end of the fiber jumper through the spectrometer (HR4000, Ocean Optics, Inc.). The spectrometer is connected to the computer by a USB port, and the home-written Labview program is used to real-time collect and process experimental data. The experimental samples are injected into a home-built flow cell by a peristaltic pump with a constant flow rate of 1 mL/min. The temperature of laboratory is always maintained at 25 °C in the following performance test, which is most suitable temperature for the sensing analysis.

**Figure 3:**
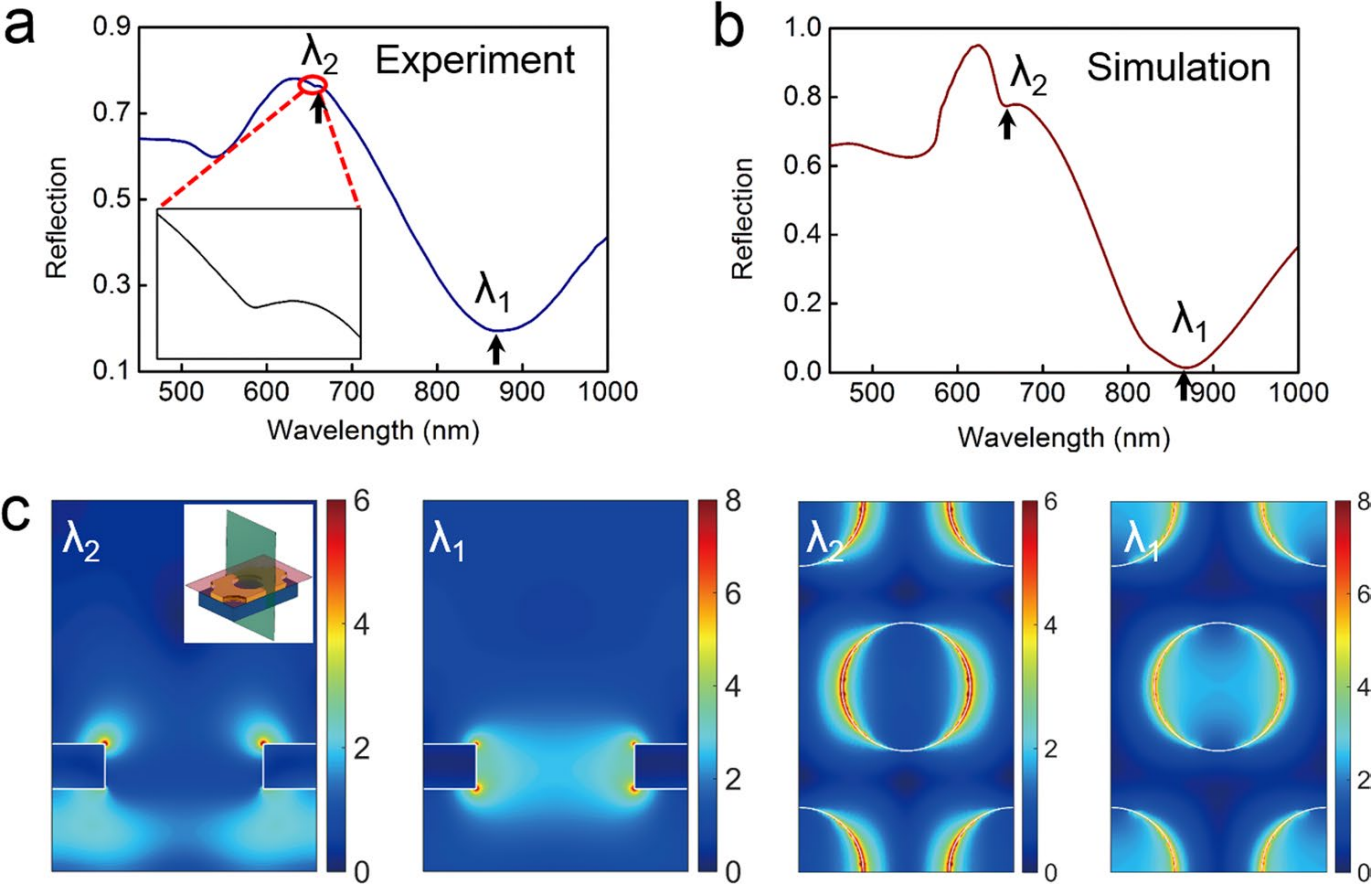
(**a**) Experimentally measured reflection spectrum of nanoholes array-based optical fiber probe measured in water and (**b**) its corresponding simulation. The reflection spectra are normalized by using gold film. The signs λ_1_ and λ_2_ respectively refer to two different plasmonic resonant modes. Dip positions are marked by black arrow. (**c**) Electric field profiles for dips λ_1_ and λ_2_ on top surface and cross-section of nanoholes array. In the inset of (**c**), the top surface is marked by a light red plane and cross-section corresponds to a dark green plane.

The normalized experimental reflection spectrum of plasmonic fiber nanoprobe in water and its corresponding simulated result are depicted in Fig. 3(a,b), respectively. The numerical simulations are performed based on finite-difference time-domain (FDTD) algorithm. The permittivity of Au film in the visible and near-infrared region is from Johnson and Christy^30^ and the refractive index of optical fiber substrate is 1.46. Experimental measured spectrum exhibits good agreement with simulated result in term of dip positions and variation tendency. There are three dips observed at the wavelengths of 868 nm (λ_1_), 657 nm (λ_2_), and 530 nm. The dip around 530 nm comes from the interband transitions of Au film and is dependent on the background reference light. To better understand the physics origins associated with the spectral features (dips λ_1_ and λ_2_) of nanoholes array, simulated electric field distributions on the top surface and cross-section of nanoholes array for dips λ_1_ and λ_2_ are shown in Fig. 3(c). For dip λ_1_, it can be seen clearly that electric field is mainly localized inside subwavelength hole and forms cavity mode in the nanohole. This cavity mode regarded as a “bridge” builds up resonant coupling between surface plasmons at the top and bottom surfaces, which leads to constructive interference in the forward direction^31^. Therefore, dip λ_1_ comes from the resonant coupling of surface plasmons at two interfaces. In contrast to dip λ_1_, the electric field of dip λ_2_ is mainly localized on the top and bottom surfaces. Strong cavity mode has not been formed inside the nanohole for dip λ_2_, which hinders efficient coupling between surface plasmon modes at top and bottom surfaces. Compared to dip λ_1_, the depth of dip λ_2_ in experiment is much smaller, which is difficult to detect and distinguish the sensing signal from background noise in experiment. Therefore, we only evaluate the sensing performances of dip λ_1_ in the following.

**Figure 4:**
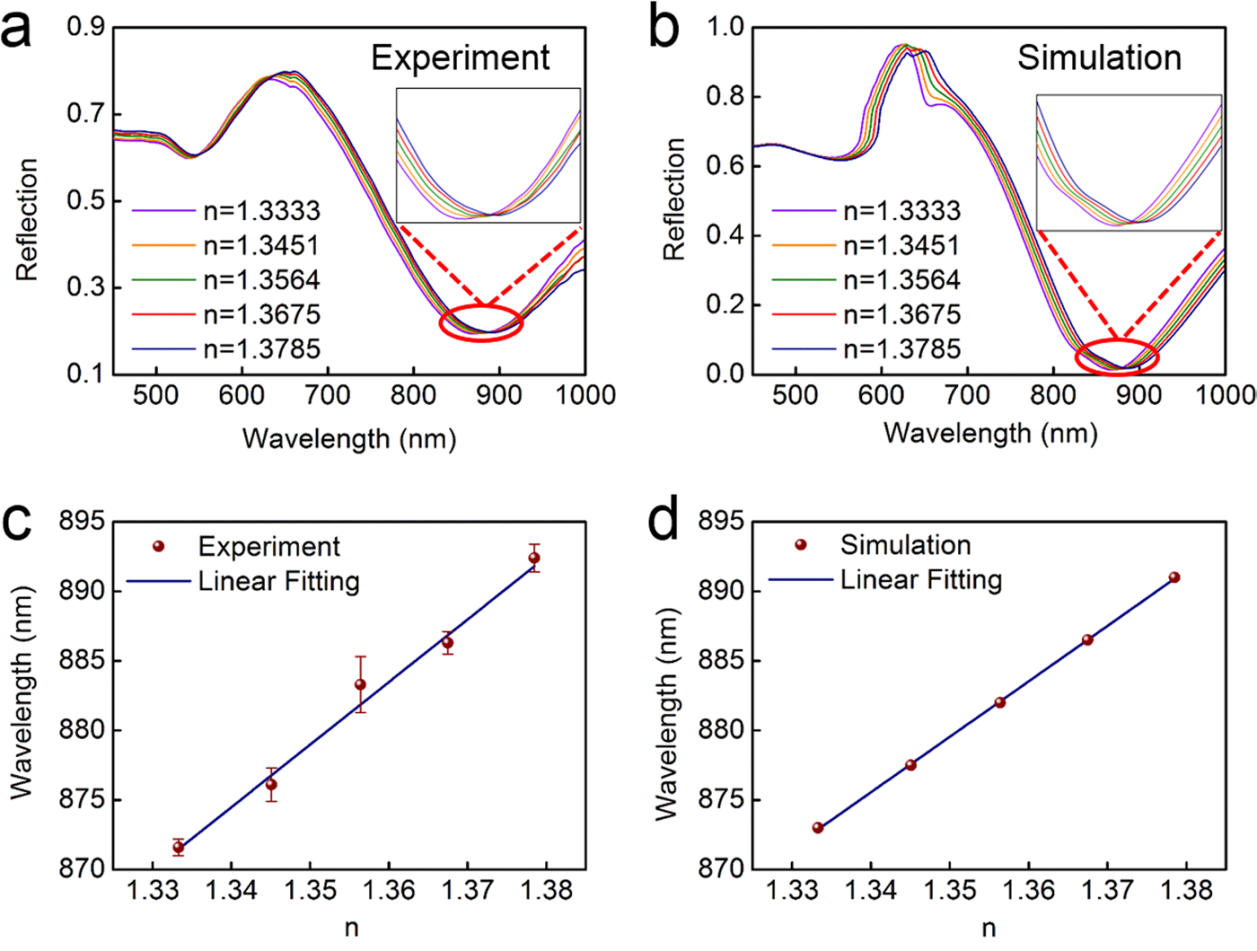
(**a**) Experimentally measured and (**b**) simulated reflection spectra of nanoholes array-based optical fiber probe in the NaCl solutions with various refractive indexes of 1.3333, 1.3451, 1.3564, 1.3675, and 1.3785. (**c**) Relationship between measured wavelength position for dip λ_1_ and the refractive index of NaCl solutions and (**d**) corresponding simulated results. The error bars in (**c**) are based on the standard deviations of three repeated measurements for every experimental data point. The blue curves in (**c**,**d**) are obtained by linear fitting.

To determine the real-time sensing performances of plasmonic fiber nanoprobe as a refractive index sensor, sodium chloride (NaCl) solutions with five refractive indexes (RIs) of 1.3333, 1.3451,1.3564, 1.3675, and 1.3785 are automatically injected into the flow cell using a peristaltic pump. The RIs of NaCl solutions are calibrated using an Abbe refractometer. The measured reflection spectra of plasmonic fiber nanoprobe with different RIs NaCl solutions are shown in Fig. 4(a). The wavelength of dip λ_1_ has an obvious red shift with the increase of the RIs of NaCl solutions. The FDTD calculated reflection spectra for an equivalent change to RIs of the NaCI solutions are shown in Fig. 4(b). Simulated results show that wavelength of dip shifts from 873 nm to 891 nm as the RIs of solutions changes from 1.3333 to 1.3785, which is in accordance with experiment results (from 871.6 nm to 892.4 nm). As shown in Fig. 4(c), wavelength change of dip λ_1_ has a linear response to the RIs of solutions. The bulk RI sensitivity (432 ± 21 nm/RIU) of nanopatterned optical fiber probe is obtained by linearly fitting experiment results with a fit coefficient *R*^2^ = 99.46%, which also agrees well with calculated RI sensitivity (401 ± 4 nm/RIU) in Fig. 4(d). Furthermore, the error bars in Fig. 4(c) indicate uncertainties of experiment measurement, which is calculated based on the standard deviations of three repeated measurements.

**Figure 5:**
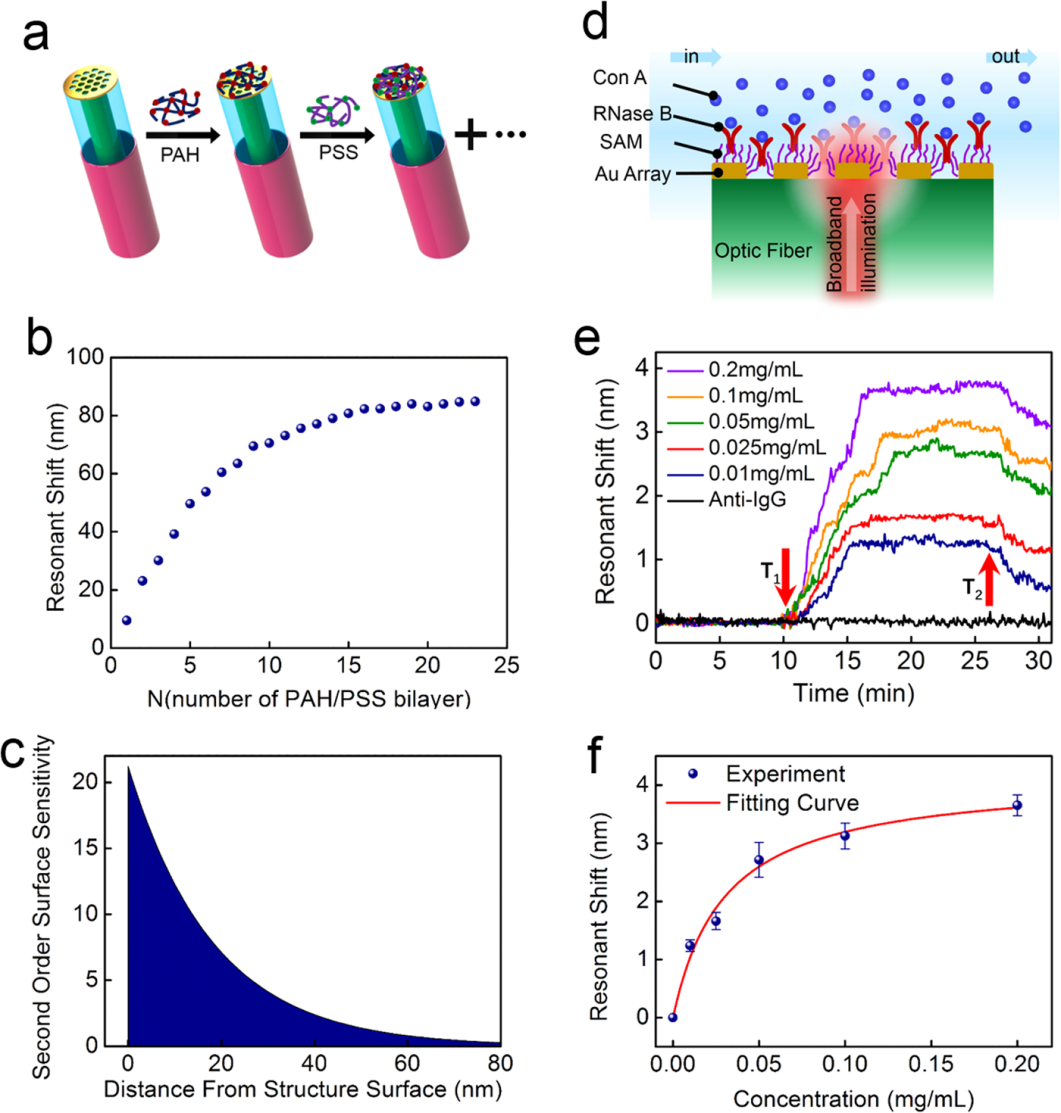
(**a**) Schematic drawing of alternating PAH/PSS bilayers absorbed on optical fiber tip by electrostatic interaction. (**b**) Wavelength shifts of resonant dip λ_1_ with the increase of PAH/PSS bilayers. (**c**) The second order surface sensitivity curves for resonant dip λ_1_. (**d**) Scheme depicting the side view of a flow channel for measuring Con A binding to RNase B immobilized on gold hole array. (**e**) Real-time resonant shift of dip λ_1_ at different concentrations of Con A. (**f**) Resonant wavelength shifts in response to specific binding of Con A with RNase B on optical fiber sensing probe for various concentrations. The error bars present the standard deviations of three repeated measurements. The red curve is fitted using the Langmuir isotherm.

Compared to conventional planar metal film, the main advantage of nanostructure used for sensing application is strong local field enhancement on the surface of structure. Therefore, the RI change of confined region around nanostructure on the sensing performance is important for biomolecule detection. Here, we use the decay length of surface electric field to characterize surface sensitivity of the plasmonic fiber nanoprobe with layer-by-layer (LBL) self-assembly technique. Figure 5(a) schematically depicts alternately self-assemble process of a positively charged poly(allylamine)hydrochloride (PAH, 1 mg/mL, 65 kDa, Sigma-Aldrich) and a negatively charged poly(styrenesulfonate) (PSS, 1 mg/mL, 75 kDa, Sigma-Aldrich) on the surface of fiber nanoprobe. The specific experimental procedures have been described in ref.^32^. As shown in Fig. 5(b), we find that the wavelength of dip has a distinct red shift with the increase of PAH/PSS bilayers. When the number of PAH/PSS bilayers increases to 16, the wavelength response of plasmonic fiber nanoprobe nearly reaches a plateau.

The results in Fig. 5(b) indicate resonant dip not only has a high surface sensitivity, but also has a strong surface electric field localization. In order to further quantitatively analyse surface sensitivity, decay length of enhanced electric field induced nanostructure surface is estimated by using the following equation^33^:

1 $${\rm{\Delta }}\lambda =m\times {\rm{\Delta }}n\times (1-{e}^{-2t/{l}_{d}})$$

Here, *m* represents bulk RI sensitivity of sensing probe; Δ*n* refers to the RI changes around nanostructure due to the absorption of polyelectrolytes; *t* is the thickness of polyelectrolytes layers; and *l*_d_ is decay length of enhanced electric field. The bulk RI sensitivity *m* and the decay length *l*_d_ can be estimated at *m* = 389 nm/RIU and *l*_d_ = 37 nm by fitting Eq. 1 to the results shown in Fig. 5(b). Compared to that of previous reported plasmonic sensor^32,34^, the decay length of resonant dip λ_1_ is much smaller, which further confirms that the plasmonic fiber nanoprobe has a high surface sensitivity. Furthermore, second order surface sensitivity is another important factor to characterize the sensing performance of nanostructure^35^. Based on the above bulk sensitivity factor *m* and the decay factor *l*_d_ in Eq. 1, Fig. 5(c) shows the second order surface sensitivity of resonant dip λ_1_. It can be clearly seen that the second order surface sensitivity of resonant mode decreases rapidly with the distance away from nanostructure surface. Small decay length and high second order surface sensitivity around nanostructure for resonant dip λ_1_ makes it attractive to detect biomolecule with small size.

To further demonstrate the sensing performance of the plasmonic fiber nanoprobe as a label-free biosensor, we use specific binding of Concanavalin A (Con A, Sigma-Aldrich) to ribonuclease B (RNase B, 0.1 mg/mL, Sigma-Aldrich) immobilized on a monolayer (11-mercaptoundecanoic acid, 2 mM, J&K Scientific)^32^ to perform protein sensitive measurement [Fig. 5(d)]. Con A, as an important member of the legume lectin family, is widely applied in biology and biochemistry to characterize glycoproteins and other sugar-containing entities on the surface of various cells^36^. The specific binding of Con A and RNase B has become a standard affinity mode for the detection of biosensor’s performances^37^. As shown in Fig. 5(e), sensorgrams are obtained by monitoring the change in the wavelength position of resonant dip λ_1_ with a time resolution of 2 s. The Con A solution is injected at time point *T*_1_ and buffer washing (phosphate buffered saline solution) is performed at time point *T*_2_. Resonant shift has an abrupt change in the sensing curves after the injection of Con A solution, which is attributed to the specific binding between Con A and RNase B. Different concentrations of Con A solution ranging from 0.01 mg/mL to 0.20 mg/mL are monitored by regenerating the biosensor surface with the urea solution (8 M, J&K Scientific). We also can see that resonant shift gradually decreases with the increase of time until zero at the washing step. Here, the urea solution is used to accelerate resonant shift restore the baseline. Moreover, the detection of protein anti-IgG in Fig. 5(e) is used as a negative control for the specific detection of optical fiber probe. It can be clearly seen that the non-specific binding between anti-IgG and RNase B occurs, which implies that good specific binding only happens between Con A and Rnase B. To validate reliability and repeatability of the optical fiber sensing probe, each concentration of Con A solution is measured repeatedly for three times. Figure 5(f) summarizes the dependence of maximal wavelength shift on the protein Con A concentration. It can be clearly seen that the wavelength shift is proportional to the protein Con A concentration at the initial region. With the increase of protein Con A concentration, most of protein RNase B sites are occupied by the protein Con A, which reaches a saturation for wavelength shift. The sensing response of the above protein molecules on the nanostructure surface can be accurately modeled by the well-known Langmuir isotherm, as shown in fitting curve of Fig. 5(f). Based on the standard deviation of experiment noise in Fig. 5(e) and the results in Fig. 5(f), the detection limit of optical fiber probe for protein Con A is about 8.1 μg/ml. Above good sensing performances demonstrated by the plasmonic fiber nanoprobe makes it be promising for the future development of a miniaturized and portable sensing platform.

## Conclusion

In summary, we demonstrate a plasmonic fiber nanoprobe which can be used as a miniaturized and portable biosensing platform. The nanoprobe is made of a multimode optical fiber integrated with Au nanoholes array, which is fabricated by low-cost self-assembly nano sphere lithography. The sensing performances, including bulk refractive index sensitivity and surface sensitivity are systematically investigated. Moreover, we also successfully perform real-time detection of biomolecular specific binding using plasmonic fiber nanprobe. This type of device is expected to bring up exciting opportunities for developing a miniaturized, integrated and portable biomedical platform.

## Experiment Method

### The self-assembly of PS nanosphere

The PS nanosphere is self-assembled at the air/water interface by using the following methods: firstly, the preparation of silicon conduit plate. The silicon plate with size of 11 × 4 cm is obtained from a standard Si wafer by diamond knife. Silicon plate is immersed successively into the acetone, chloroform, alcohol and deionized water solutions and sonicated in an ultrasound bath for 5 mins to obtain clean silicon surface. Next, the clean silicon plate is deal with Piranha solution (3:1 H_2_SO_4_/H_2_O_2_) for 30 mins to increase the hydrophilicity of the silicon surface, which is beneficial to the formation of large-scale nanosphere monolayer at the air/water monolayer. Secondly, the self-assembly of PS nanosphere at the air/water interface. The 5%wt PS nanosphere water solution is mixed the same volume of ethanol solution. In order to secure uniform mixture, the mixed solution is sonicated in an ultrasound bath for 30 mins at the temperature of 25 °C. The temperature of ultrasound bath is controlled by water reuse system. The prepared silicon plate is fixed on the edge of a glass beaker filled with ultrapure water. To increase the combination between particles, an 80 μL twelve alkyl sulfate solution (5%wt) is firstly added to water solution for changing the tension of the water surface by microsyringe. Then the nanosphere suspension solution is released along the silicon conduit plate at the rate of 2 μL/min approximately. As soon as reaching the water, the nanosphere suspension solution rapidly spreads on the surface of the water. The PS nanosphere is self-assembled in a closed-packed arrangement and forms a floating PS nanosphere monolayer at the air/water interface. The self-assembly PS nanosphere monolayer appears green because of light diffraction on the air/water interface. After the PS nanosphere monolayer is formed at the air/water interface, the self-assembly PS nanosphere monolayer is transferred from water by gently lifting it towards the floating monolayer on the surface using tweezers. The nano-patterned optical fiber is then dried in air for 24 h.
